# Late capsular bag contraction and intraocular lens subluxation in retinitis pigmentosa: a case report

**DOI:** 10.1186/1752-1947-5-65

**Published:** 2011-02-14

**Authors:** Dany M Najjar, Ann O Igbre, Frank F Tsai

**Affiliations:** 1 Temple University Hospital Department of Ophthalmology 3401 N. Broad Street Philadelphia, PA 19140, USA

## Abstract

**Introduction:**

Retinitis pigmentosa is clinically characterized by loss of predominantly rod photoreceptor function as well as loss of peripheral vision. The classic clinical triad is considered to be the presence of bone spicule pigmentation in the peripheral retina, arteriolar attenuation, and waxy disc pallor. Cataracts, most commonly of the posterior subcapsular type, are often found in all forms of retinitis pigmentosa. Ectopia lentis and lens dislocation are known risk factors for those with retinitis pigmentosa, presumably secondary to zonular fiber weakness and vitreous degeneration. The post-operative complication of lens dislocation following cataract extraction in patients with retinitis pigmentosa has also been documented.

**Case presentation:**

We report a case of severe capsular bag contraction with intraocular lens subluxation following cataract extraction in a 58-year-old Hispanic woman with retinitis pigmentosa.

**Conclusion:**

Patients with retinitis pigmentosa undergoing cataract surgery should be notified of this potentially late complication of surgery.

## Introduction

Retinitis pigmentosa (RP) is clinically characterized by loss of predominantly rod photoreceptor function as well as loss of peripheral vision. The classic clinical triad is considered to be the presence of bone spicule pigmentation in the peripheral retina, arteriolar attenuation, and waxy disc pallor. Cataracts, most commonly of the posterior subcapsular type, are often found in all forms of retinitis pigmentosa. Ectopia lentis and lens dislocation are known risk factors for those with RP, presumably secondary to zonular fiber weakness and vitreous degeneration [1,2]. The post-operative complication of lens dislocation following cataract extraction in RP patients has also been documented [2].

In this report, we describe a spontaneous late posterior chamber intraocular lens subluxation secondary to severe capsular bag contraction in a patient with RP.

## Case presentation

A 58-year-old Hispanic woman presented to our clinic with blurred vision. She had been diagnosed with RP two years prior to presentation. Her best corrected visual acuity (BCVA) was 20/70 in the right eye and 20/25 in the left. Slit-lamp examination revealed anterior subcapsular as well as nuclear cataracts in both eyes, but worse in the right eye. No phacodonesis or iridodonesis was noted. Dilated fundus examination revealed bone spicule pigmentation in the retinal periphery, arteriolar attenuation, and optic disc pallor in both eyes, but which was more prominent in her right eye than in her the left. She had severe field loss of peripheral visual in the right eye (measured by Humphrey visual field. Humphrey Field Analyzer, Zeiss Ophthalmic, Dublin, CA, USA), leaving only the central 10°. Her left eye was found to have only mild loss of peripheral vision nasally. Electroretinography demonstrated an isoelectric potential in her right eye consistent with the diagnosis of RP. In her left eye, there was a generalized decrease in amplitude. She underwent an uncomplicated cataract extraction by phacoemulsification of the right eye. A continuous curvilinear capsulorrhexis was performed without any zonular stress observed. The capsulorrhexis was approximately 6 mm in diameter. A foldable acrylic intraocular lens (Tecnis Abbott Medical Optics Inc. Santa Ana, CA, USA) was inserted and observed to be well-centered at the end of the case.

**Figure 1:**
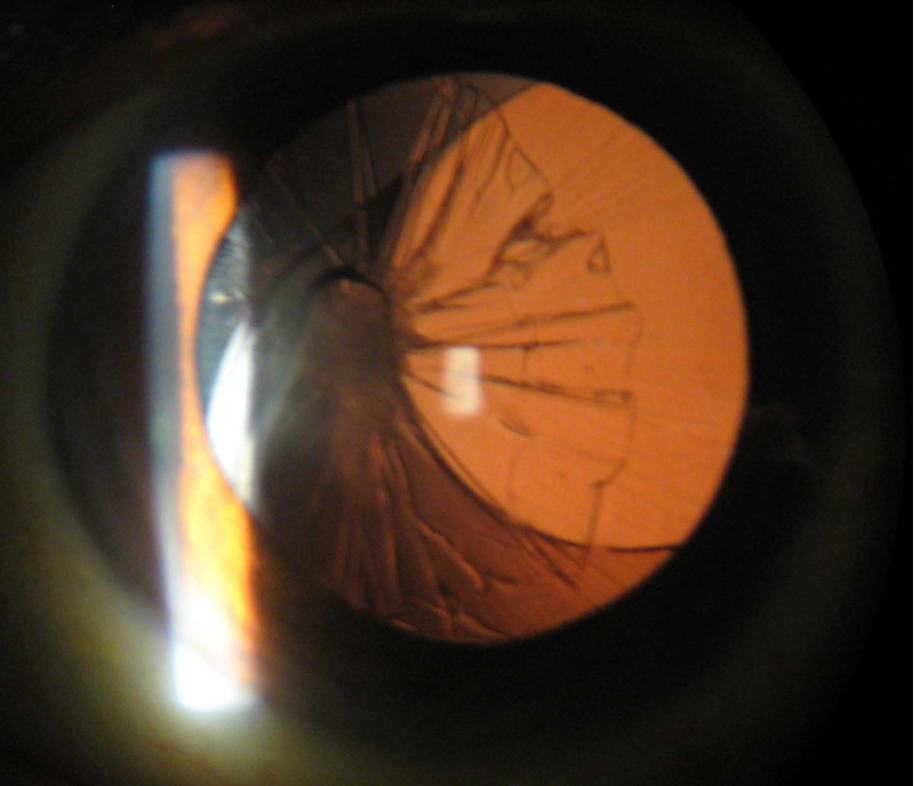
**Capsular bag contraction and intraocular lens subluxation**.

Post-operatively, her BCVA improved to 20/40 in the right eye. The intraocular lens was well-positioned within the capsular bag. However, three months after the surgery, she again presented to the clinic with blurry vision. She denied any history of trauma or fall. At this time her BCVA was 20/100 in the right eye and 20/25 in the left eye. On slit-lamp examination, the edge of the intraocular lens was displaced nasally and the capsular bag was displaced temporally (Figure 1). There were prominent capsular bag folds indicating severe bag contraction. Fundus examination again revealed changes from RP that were stable compared to previous examination. She was offered surgery, but declined it

## Discussion

There is a strong association between RP and zonular fiber weakness, anterior capsule contraction, and extensive vitreous degeneration [2,3]. In one case study of a Korean patient with RP, intraocular lens dislocation into the anterior chamber occurred six years after phacoemulsification of the left eye and eight years after extracapsular cataract extraction of the right eye [4]. Hayashi, in a 2007 study, documented the possible predisposing factors for late in-the-bag or out-of-the-bag intraocular lens dislocation after intraocular lens placement [3]. RP was found to be associated with an increased incidence of in-the-bag lens dislocation. One case study reported a posterior lens dislocation in an RP patient one year following posterior Nd-YAG laser capsulotomy [5].

Although there is the possibility of zonular disruption having been inflicted during surgery in our patient, the lens was well-positioned and stable for more than 10 weeks post-operatively. Nonetheless, it is likely that the turbulent forces of the phacoemulsification process further disrupted an already weak set of zonular fibers. Dehiscence of these fibers and the fibrotic changes found in the anterior capsule are likely to have contributed to the development of severe capsule contraction and intraocular lens subluxation in this patient.

Again, it is possible that a tear in the posterior capsule was created during surgery or from asymmetrical placement of the intraocular lens. However, no tear was detected visually and there was no prolapse of vitreous into the posterior chamber. Additionally, the correct placement of the intraocular lens was confirmed intra-operatively as well as on multiple post-operative visits. Thus, severe capsular contraction combined with anterior vitreous degeneration and zonular fiber weakness provides the most plausible mechanism of lens subluxation.

## Conclusion

Although the occurrence of posterior lens subluxation following phacoemulsification and intraocular lens placement is not a common event [3], it is a complication that should be discussed with patients with RP undergoing cataract surgery given its ability to cause dramatic consequences. Furthermore, careful slit-lamp examination is essential preoperatively to detect any zonular weakness or loss. If this weakness is present, consideration should be given as to the method of cataract extraction that would best preserve zonule fiber integrity and limit the risk of posterior capsule tears. For example, a chopping technique may be preferred to reduce zonular stress. In addition, gentle hydrodissection can also be employed to decrease stress on the zonules during surgery. Consideration should be given to using a capsular tension ring prophylactically in the fellow eye in order to help prevent a similar event.

## Consent

Written informed consent was obtained from the patient for publication of this case report and accompanying images. A copy of the written consent is available for review by the Editor-in-Chief of this journal.

## Competing interests

The authors declare that they have no competing interests.

## Authors' contributions

DN gathered the data, performed the literature review, and edited the manuscript. FT and AI were major contributors in writing the manuscript. All authors read and approved the final manuscript.
